# Laboratory Predictors of Prognosis in Cardiogenic Shock Complicating Acute Myocardial Infarction

**DOI:** 10.3390/biomedicines10061328

**Published:** 2022-06-05

**Authors:** Tamilla Muzafarova, Zuzana Motovska

**Affiliations:** Cardiocenter, Third Faculty of Medicine Charles University and University Hospital Kralovske Vinohrady, 10034 Prague, Czech Republic; muzafarovat@fnkv.cz

**Keywords:** acute myocardial infarction, cardiogenic shock, outcomes, laboratory biomarkers

## Abstract

Cardiogenic shock is a state of reduced cardiac output leading to hypotension, pulmonary congestion, and hypoperfusion of tissues and vital organs. Despite the advances in intensive care over the last years, the morbidity and mortality of patients remain high. The available studies of patients with cardiogenic shock suggest a connection between clinical variables, the level of biomarkers, the results of imaging investigations, strategies of management and the outcome of this group of patients. The management of patients with cardiogenic shock initially complicating acute myocardial infarction is challenging, and the number of studies in this area is growing fast. The purpose of this review is to summarize the currently available evidence on cardiogenic shock initially complicating acute myocardial infarction with particular attention to predictors of prognosis, focusing on laboratory variables (established and new), and to discuss the practical implementation. Currently available scoring systems developed during the past few decades predict the clinical outcome of this group of patients using some of the established biomarkers among other variables. With the new laboratory biomarkers that have shown their predictive value in cardiogenic shock outcomes, a new design of scoring systems would be of interest. Identifying high-risk patients offers the opportunity for early decision-making.

## 1. Introduction

Cardiogenic shock (CS) is characterized by low cardiac output resulting in life-threatening target organ hypoperfusion and tissue hypoxia. 

The most common cause of CS is acute myocardial infarction (AMI) (in 81% of cases) [1]. Approximately 5 to 8% of ST-elevation myocardial infarction (STEMI), and 2 to 3% of non-ST-elevation myocardial infarction (NSTEMI) can result in CS. Close to 45% of patients with CS complicating AMI initially die despite optimal treatment [2].

There are few definitions of CS presented by modern guidelines and trials. The European Society of Cardiology guidelines 2021 define CS on the base of hypotension—a systolic blood pressure under 90 mmHg with appropriate fluid resuscitation with clinical (cold sweated extremities, oliguria, mental confusion, dizziness, narrow pulse pressure) and laboratory (elevated serum lactate and creatinine, metabolic acidosis) signs of hypoperfusion [3]. The SHOCK (Should We Emergently Revascularize Occluded Coronaries for Cardiogenic Shock) trial used either clinical criteria (hypotension—a systolic blood pressure under 90 mmHg for at least 30 min or above 90 mmHg with supportive measures, signs of end-organ hypoperfusion (cool extremities or a urine output of <30 mL per hour, and a heart rate of ≥60 beats per minute) or hemodynamic criteria (cardiac index of no more than 2.2 L/min/m^2^ and a pulmonary-capillary wedge pressure of at least 15 mmHg) [4]. According to IABP-SHOCK II (Intraaortic Balloon Pump in Cardiogenic Shock II) randomized trial, the diagnostic criteria included at least one sign of impaired end-organ perfusion (altered mental status; cold, clammy skin and extremities; oliguria < 30 mL/h; or serum lactate ≥ 2.0 mmol/L) [5].

In general, in the pathogenesis of cardiogenic shock complicating acute myocardial infarction (CS-AMI), impaired myocardial contractility results in a low cardiac output and low blood pressure leading to systemic hypoperfusion, ischemia of organs, inclusion of inflammatory mediators, vasoconstriction, volume overload resulting in multiorgan system failure and death, if not adequately treated in time [6]. The Society of Coronary Angiography and Intervention has published a clinical expert consensus statement on the classification of cardiogenic shock, where cardiogenic shock is divided into the following stages—at risk, beginning, classic, deteriorating and extremis [7]. This classification demonstrates that patients have different degrees of clinical and hemodynamic state and are indicated for different therapeutic strategies. 

Thus, the study of factors affecting the course and outcome of the cardiogenic shock, including clinical, laboratory and imaging parameters and early identifying patients at the highest risk is essential. Due to the high mortality rate, research in this area is enormous and the number of published studies is growing fast. We summarized and presented the available studies in this field with the aim of an up-to-date overview.

## 2. Materials and Methods

The bibliography for this study was compiled until March of 2022 through a literature search using the PubMed search engine without limits set on publication status or start date. A systematic search included all articles reviewing laboratory predictors of prognosis of CS exclusively caused by AMI. Studies with an etiology of CS other than AMI were not considered. The references cited in the selected articles were also reviewed for additional references.

## 3. Results

### 3.1. Creatinine Clearance

Renal function is an important clinical sign of outcome prediction in patients with CS-AMI. According to the IABP-SHOCK II (Intraaortic Balloon Pump in Cardiogenic Shock II) randomized trial substudy, serum creatinine level was shown as a significant independent predictor of 1-year mortality in a multivariable analysis compared to glomerular filtration rate [8]. In the multicenter randomized clinical trial TRIUMPH (Tilarginine Acetate Injection in a Randomized International Study in Unstable MI Patients With Cardiogenic Shock) creatinine clearance along with systolic blood pressure, measured on vasopressor support, were significant predictors of mortality in all models, creatinine clearance per 10 mL/min increase was a significant univariably associated predictor of 30-day mortality (odds ratio (OR) 0.77; 95% confidence interval (CI) 0.7-0.84; *p* < 0.001) [9]. Several other studies showed the predictive value of renal function as well. The American College of Cardiology–National Cardiovascular Data Registry of 483 patients aged 65 ± 13 years who underwent percutaneous coronary intervention (PCI) in the setting of CS-AMI identified serum creatinine level above 2.0 mg/dL (OR 4.69; 95% CI 1.96-11.23; *p* < 0.001) among five other multivariate predictors of death [10]. In the study of 2020 patients with STEMI who underwent primary PCI within 12 h after symptom onset, creatinine clearance < 60 mL/min in 141 patients presented with CS on admission was identified as one of independent predictors of 30-day mortality (hazard ratio (HR) 2.75; 95% CI 1.66–4.66; *p* < 0.0001) [11]. The role of creatinine clearance in outcome prediction of CS-AMI patients was highlighted in a number of score systems (Table 1). The score system based on the SHOCK (Should We Emergently Revascularize Occluded Coronaries for Cardiogenic Shock) trial and registry through multivariable modeling has identified eight risk factors predicting 30-day in-hospital mortality risk, among them is creatinine level ≥ 1.9 mg/dL (c-statistic = 0.74) [12]. The IABP-SHOCK II trial used creatinine level at admission >1.5 mg/dL among six other variables predicting a 30-day mortality risk (HR 1.57; 95% CI 1.17–2.11; *p =* 0.003) [13]. 

Acute kidney injury (AKI) is a frequent clinical complication of CS-AMI and is a strong predictor of mortality, as the kidneys receive 20 to 25% of the cardiac output [18]. According to KDIGO (The Kidney Disease: Improving Global Outcomes) guidelines, AKI is defined as an increase in creatinine level above 0.3 mg/dL or above 50% from baseline [19]. In a single-center study of 97 consecutive patients with STEMI complicated by cardiogenic shock at admission, patients with AKI (with rise in creatinine level above 25% from baseline) had a poor prognosis and significantly higher mortality rate (50% vs. 2.2%; *p* < 0.001) compared to patients without AKI [20].

**Figure 1:**
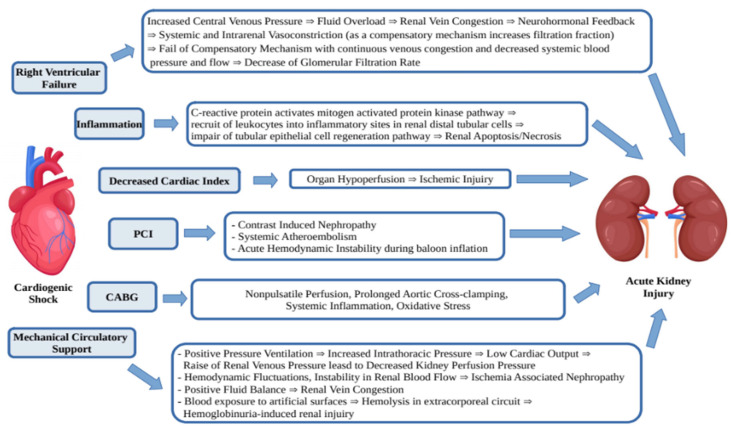
Mechanisms of acute kidney injury in CS-AMI.

The AKI in patients with CS-AMI develops due to a few mechanisms (Figure 1). It is known that acute decrease of blood flow and end-organ hypoperfusion leads to oliguria, but some other mechanisms such as right ventricular failure, inflammation, systemic and venous congestion, PCI, CABG and mechanical circulatory support play an additional role [21].

**Figure 2:**
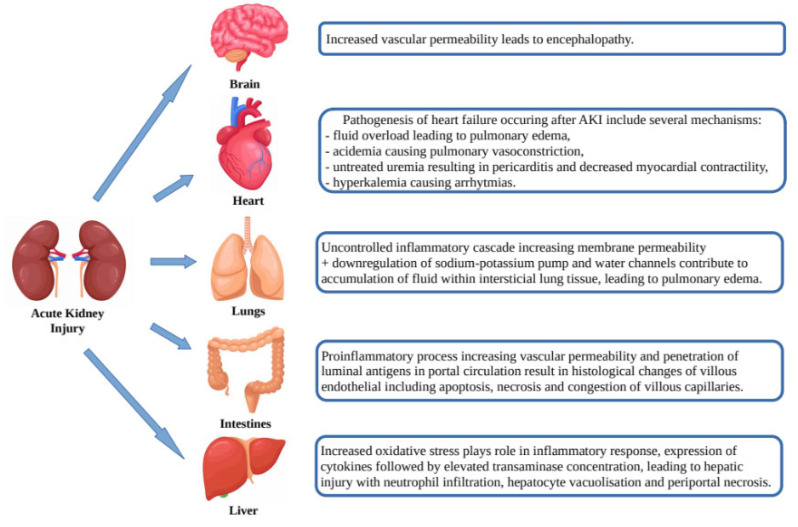
Effects of acute kidney injury on organ dysfunction.

AKI is not only the result of the abovementioned pathogenic pathways, but itself may affect organ dysfunction (heart, lungs, brain, intestines and liver) through a number of mechanisms such as release of cytokines, oxidative stress, leukocyte extravasation and Na+ and H_2_O channel dysregulation leading to venous congestion, vascular permeability, apoptosis/necrosis and organ dysfunction (Figure 2) [22].

Multiple CS-AMI risk scores identified chronic kidney disease as an important predictor of poor prognosis. Thus, as shown above, creatinine reflecting a worsening of renal function during admission for CS-AMI may be used as a powerful predictor of outcomes and mortality in this group of patients. However, there are some limitations and the levels of baseline creatinine are not available in some cases. 

### 3.2. Glucose Serum Levels

The correlation between plasma glucose levels and outcomes of CS-AMI has been investigated in a number of studies. The analysis of 7431 Chinese STEMI patients from the CREATE (Clinical Trial of Reviparin and Metabolic Modulation in Acute Myocardial Infarction Treatment Evaluation) trial showed seven independent factors of 30-day mortality, including admission glycemia above 7.8 mmol/L (OR 2.17; 95% CI 1.26–3.73) along with age, anterior infarction, serum sodium levels under 130 mmol/L, left ventricular ejection fraction < 40% and no emergency revascularization [23]. In the study of D. Pres et al. [24] including 258 patients with CS-AMI, the multivariate analysis showed that, regardless of the history of diabetes mellitus, the admission level of blood glucose above 7.8 mmol/L was an independent factor of in-hospital (OR 1.08; 95% CI 1.02–1.14; *p* = 0.0044), 1-year (HR 1.04; 95% CI 1.01–1.06; *p* = 0.005) and 5-year mortality prediction ( HR 1.03; 95% CI 1.01–1.05; *p* = 0.045). In the other study of 208 patients with CS initially complicating STEMI and without history of diabetes, multivariate analysis showed a 16% mortality increase for every 1 mmol/L increase in plasma glucose concentration (OR 1.155; 95% CI 1.070–1.247), after adjustment for age, sex, left ventricular ejection fraction and TIMI flow after PCI [25]. 

Increased glycemia has been shown to have a harmful effect on cardiomyocytes, which may be explained by such effects of hyperglycemia as metabolic derangement accompanying hyperglycemia, altered platelet metabolism, intraplatelet signaling pathway changes, promotion of inflammatory cascade, impaired perfusion, impaired left ventricular function, Table 2.

Hyperglycemia may induce electrophysiologic alterations in AMI and favor the occurrence of fatal arrhythmias [26]. Marfella et al. reported significant QT prolongation in patients when plasma glucose concentration was rapidly increased to 15 mmol/L [27]. As it was shown in a study of 146 patients with AMI, a no-reflow phenomenon was observed in patients with hyperglycemia on hospital admission, so hyperglycemia may be associated with disorders of coronary microcirculation, larger size of infarct, and thus poor outcome [28]. Insulin injection in this group of patients may provide metabolic control by inhibiting lipolysis, reducing the level of free fatty acids and improving the use of myocardial glucose and leading to a better long-term prognosis. This idea was studied by Malmberg et al. in 620 diabetic patients with AIM, divided into a group of 306 patients who received intensive insulin treatment and a control group of 314 patients. The results have shown one-year mortality rate 18.1% in the insulin group compared to 26.1% in control group, this corresponds to a relative reduction in one-year mortality of 29% (*p* = 0.0273) [29,30].

### 3.3. Lactate Blood Concentration

Lactate as a metabolic product of anaerobic glycolysis reflects inadequate oxygen delivery and could be used as a marker of tissue perfusion. Besides being a marker of hypoperfusion, lactate presents an alternative energy source for patients in cardiogenic shock. Lactate is produced in the citric acid cycle from the degradation of pyruvate and is mainly metabolized in the liver and kidneys to restore energy resources. The heart mainly metabolizes free fatty acids and pyruvate, but 20% of the heart’s energy needs are covered by lactate oxidation. In physical activity, lactate oxidation increases due to enhanced stimulation of beta-adrenergic receptors and increased concentration of arterial lactate [31].

In cardiogenic shock, reduced cardiac output leads to hypoperfusion of tissues and microvascular disorders, involving inflammatory markers and catecholamines, contributing to high lactate production. Stress induces sympathoadrenal activation, stimulation of beta-2 receptors increases aerobic glycolysis in heart, in addition to accelerated anaerobic lactate production. Tissue hypoperfusion is accelerated furthermore by hypotension and reduced left ventricular ejection fraction, thus leading to an increase of anaerobic lactate production [32]. Thus, lactate appears to be a marker of hypoperfusion and adrenergic stress [32].

A number of studies have shown the connection between elevated blood concentrations of lactate and worse outcome in patients with CS. The secondary analysis of the CardShock study including 219 patients with CS-AMI reported lactate as a strong predictor of 30-day mortality (HR 1.20; 95% CI 1.14–1.27), and a relative change in lactate concentration as a significant survival predictor during the first 24 h of intensive treatment [33].

In a study of 45 patients with CS-AMI who underwent primary PCI, a multivariate analysis reported the blood concentration of lactate above 6.5 mmol/L as an independent indicator of in-hospital mortality (OR 295; 95% CI 3.4–25444; *p* = 0.01) after adjustment for sex, age, hypertension and diabetes, and a univariate predictor of in-hospital mortality (OR 54; 95% CI 5.8–494.9; *p* < 0.0001) along with another six factors (age, arterial hypertension history, blood concentrations of glucose and uric acid, TIMI flow after PCI) [34]. The prognostic significance of lactate level was studied by Koreny et al., who reported that in patients with developed AKI (oliguria < 20 mL/h and creatinine >50% above the baseline value) within the first 24 h after CS-AMI onset, the level of lactate along with creatinine and epinephrine dose was a strong univariate predictor of in-hospital mortality, but the multivariate analysis of this study has identified AKI as the only independent mortality predictor [35].

The reduction of lactate blood concentration reflecting improved organ perfusion could be used in evaluation of treatment and prognosis as well. The Impella-EURO SHOCK registry which analyzed the association between mortality in patients with CS-AMI and the changes of plasma lactate levels within 24 and 48 h after hemodynamic support identified the admission level of lactate above 3.8 mmol/L as a 30-day mortality predictor (OR 5.245; 95% CI 1.473–18.677; *p* = 0.011) [36].

The number of studies presented above confirm the prognostic effect of elevated serum lactate, but the cut-off values of lactate indicating poor prognosis have not been defined yet [37]. It would be of interest to conduct further investigations for defining the cut-off values for hemodynamic management strategy. Table 3 presents studies reviewing the prognostic effects of lactate blood concentration.

Recent studies show that a higher lactate clearance after treatment initiation of patients with CS-AMI have a high association with a more favorable outcome. In patients with CS-AMI, 12 h lactate clearance under 10% is an indicator of short and long-term mortality risk (*p* = 0.002) [38]. The systemic review of 12 studies analyzing the association between lactate clearance and outcomes in patients with CS showed that survivors had 17.3% higher level of lactate clearance at 6–8 h (*p* < 0.001) and 27.9% higher level of lactate clearance at 24 h (*p* < 0.001) compared to non-survivors [39]. In a randomized, double-blind, controlled DOREMI (Dobutamine Compared to Milrinone in the Treatment of Cardiogenic Shock) prospective trial, lactate clearance was a significant survival predictor at all time points, with OR between 2.46 at 8 h (95% CI 1.09–5.55; *p* = 0.03) to 5.44 at 24 h (95% CI 2.14–13.8; *p* < 0.01) [40]. The possible pathologic mechanisms of the above mentioned association was investigated in the recent Cardiac Magnetic Resonance Imaging Study in 360 patients with AMI undergoing PCI, where the levels of admission lactate above 2.5 mmol/L showed a higher association with a larger size of myocardial injury (OR 1.59; 95% CI 1.00–2.51; *p* = 0.048) [41]. 

### 3.4. Hemoglobin Levels

The association of hemoglobin levels and the risk of in-hospital cardiac arrest was elucidated upon by a number of studies. The multivariate analysis of a retrospective study of 211 consecutive patients with CS-AMI showed hemoglobin concentration under 112 g/L as an independent strong predictor of in-hospital cardiac arrest; in contrast, a 1-g/L increase in the hemoglobin levels indicated a 2.9% lower risk [42]. Therefore, the monitoring of hemoglobin levels could facilitate an early decision process. High hemoglobin levels, in contrast, may serve as a protective factor against in-hospital cardiac arrest. This study was the first to show this correlation. The lower hemoglobin levels are more often observed in elderly patients, who are more fragile and have more comorbidities, which may be one of the reasons for this correlation. The question is if this association is higher in younger patients. Underlying pathophysiological mechanisms were summarized by Xu et al. Patients with anemia are more likely to develop ventricular arrhythmias, anemia induces tissue hypoxia in the ischemic regions and increases myocardial workload [42]. 

### 3.5. Hypoalbuminemia

Hypoalbuminemia is associated with a worse outcome and higher mortality of patients with CS after AMI. A prospective multinational study on cardiogenic shock including 178 patients was the first to assess the prognostic value of hypoalbuminemia (<34 g/L), the decrease of plasma albumin concentration measured 0–12 h has shown a linear association with increased mortality, but no association with 90-day mortality was shown [43]. Thus, plasma albumin level could be evaluated in the early stages of CS-AMI. A few reasons may possibly explain these findings. In CS, higher levels of inflammation lead to the leak of albumin. Furthermore, hypoalbuminemia may be associated with other comorbidities and the frailty of the patient. However, it could not be detected whether hypoalbuminemia was pre-existing in the studied group of patients, or the status of fluid resuscitation, in which hemodilution could be the reason of hypoalbuminemia. 

### 3.6. N-Terminal Pro-Brain Natriuretic Peptide

The N-terminal fragment of the prohormone of the brain natriuretic peptide, which is synthesized in the ventricular myocardium, reflects the level of cardiac stretch, serving as a marker of a hemodynamic stress. As reported in a number of studies, natriuretic peptides have been proven to show association with outcome of patients with CS-AMI. 

The number of affected coronary vessels, degree of stenosis and proximal left anterior descending artery disease were significantly higher in those with high NT-proBNP (≥474 pg/mL), TIMI flow grade was significantly higher in those with low NT-proBNP (<474 pg/mL) [44]. Interestingly, although long-term (6 month and 1 year) survival of patients with CS-AMI is higher with early infarct-related artery revascularization, the 30-day mortality after successful PCI differed significantly depending on levels of Nt-pro-BNP and IL-6 [4]. 

Both multivariate and univariate survival analysis of the retrospective study of 58 patients with CS-AMI showed NT-pro-BNP levels above 12,782 pg/mL as a strong 30-day mortality predictor (OR 86.2; 95% CI 74–99; *p* < 0.001) and a complementary role with interleukin-6 (IL-6) in outcome prediction was detected [45]. The study of 438 patients with STEMI up to 6 h after onset reported BNP level above 80 pg/mL as one of the strong mortality risk predictors (OR 7.2; 95% CI 2.1–24.5; *p* = 0.001) [46]. Cardiac cell wall stretching increases both during acute ischemic insult and later as a result of infarction. The importance of NTproBNP is valuable as it not only reflects the area of damage of the myocardium but the area of myocardial ischemia without infarction as well.

### 3.7. Systemic Inflammation

Most patients with CS have experienced systemic inflammation with inappropriate vasodilatation, possibly contributing to an excessive mortality rate [47]. Furthermore, in later stages of shock, decreased systemic vascular and vasopressor resistance is found during the inflammatory process. How these pathways are mediated is not well understood [48]. Systemic inflammation observed in patients with AMI and CS is accompanied by leukocytosis and high levels of acute-phase reactants. The release of such inflammatory mediators such as IL-6 and tumor necrosis factor-alpha (TNF-alpha) leads to systemic inflammatory response syndrome. It has been suggested that CS causes the release of these very factors and is possibly associated with impaired survival rates even after early revascularization [49].

Some studies have examined the relationship between the proinflammatory cytokine IL-6, CS, and multi-organ failure. A. Geppert et al. have reported that IL-6 is exhibited in a similar magnitude when comparing patients with septic shock and patients with CS: a high level of IL-6 was an indicator of higher risk of progression to multi-organ failure [50]. The analysis of 38 patients with CS-AMI showed the IL-6 level as a specific and sensitive marker of 30-day mortality with HR 1.49 (95% CI 1.24–1.80) per 50 mg/mL increase of IL-6 [51]. Thus, IL-6 could be used as an early determinant of future multi-organ failure and as a 30-day mortality prognostic marker. Other proinflammatory cytokine levels, such as IL-8, IL-10, IL-7, are associated with increased mortality rates. In the IABP-SHOCK II trial, the inflammatory marker substudy reported an association of higher levels of IL-8 and IL-10, and lower IL-7 levels with the mortality risk [52]. 

The IABP-SHOCK II trial biomarker substudy showed the correlation of cytokine interferon-γ (INF-γ), tumor necrosis factor-α (TNF-α), macrophage inflammatory protein-1β (MIP-1β), granulocyte-colony stimulating factor (G-CSF), and monocyte chemoattractant protein-1β (MCP-1β) with higher mortality risk to patients with CS-AMI [53]. 

The abovementioned studies investigating the prognostic value of inflammatory markers highlighted the potential effect of anti-inflammatory interventions for the prevention of fatal outcomes of CS.

### 3.8. Novel Biomarkers

#### 3.8.1. Activated Protein C

Another study investigating the role of inflammatory mechanisms in the outcomes of CS-AMI has detected lower levels of activated protein C (aPC) in patients who did not survive up to 28 days. Furthermore, patients with lower aPC levels were in higher need of vasopressor support [54]. 

#### 3.8.2. Catalytic Iron

Another IABP-SHOCK II trial biomarker substudy suggested that short-term mortality in CS-AMI may be associated with the catalytic iron (CI) blood concentration [55]. Catalytic iron is the oxidized form of ferric iron, which plays the role of catalyst in the Fenton and Haber–Weiss reactions, resulting in reactive oxygen species production. The high level of catalytic iron stimulates free hydroxyl radical release, which may cause endothelial apoptosis and lead to vascular injury [55]. It should also be noted that levels of CI can be influenced by mechanical resuscitation, bleeding, and myocardial necrosis [55]. The research focused on the prognostic effects of CI in patients with CS after AMI at relatively early stage.

#### 3.8.3. Osteoprotegerin and Growth Differentiation Factor 15

Osteoprotegerin (OPG) is a cytokine of the tumor necrosis factor family, expressed by osteoblasts, epithelial cells, vascular endothelial cells, B-cells and dendritic cells of the immune system. OPG plays a role in myocardial reperfusion injury, stimulating migration of leukocytes in the coronary artery wall [56]. As an indicator of acute heart injury, OPG may play a role in the reperfusion disorder by itself, thus influencing the outcome of patients with CS-AMI. 

GDF-15 is a member of the transforming growth factor beta superfamily. The high prognostic role of it in heart diseases has been actively studied in the last decade. GDF-15 production is promoted by oxidative stress, inflammation, ischemia and organ damage, observed in cardiogenic shock, which explains the predictive role of GDF-15 in CS-AMI outcome prediction. 

The IABP-SHOCK II Trial biomarker substudy has reported a higher 30-day mortality rate in patients with levels of GDF-15 above 7662 ng/L (HR 1.88; 95% CI 1.21–2.94; *p*= 0.005) and OPG above 626 ng/L (HR 1.74, 95% CI 1.11–2.71; *p* = 0.01) [57]. In a multivariate analysis, GDF-15 is a strong predictor of 30-day mortality along with age, serum lactate level, left ventricular ejection fraction and post-PCI TIMI flow grade under 3 [57]. Another study of patients undergoing PCI within 12 h after STEMI symptoms onset defined OPG as an independent predictor of major adverse cardiovascular events and shows correlation with larger cardiac injury [58].

#### 3.8.4. Fibroblast Growth Factor 23

Fibroblast growth factor 23 (FGF23) is a member of the FGF family and participates in phosphate and vitamin D metabolism and regulation. In kidneys, the FGF-23 binds to the fibroblast growth receptor and to the coreceptor Klotho suppressing renal phosphate reabsorption and circulating levels of 1.25-dihydroxyvitamin D. The IABP-SHOCK II trial substudy reported an elevated level of FGF-23 as an independent marker of 30-day (OR 1.80; 95% CI 1.11–2.92; *p* = 0.02) and 1-year mortality (HR 1.5; 95% CI 1.11–2.04; *p* = 0.009) in CS-AMI patients [59]. According to Faul et al., FGF-23 correlates with left ventricular hypertrophy development in vivo and in vitro [60]. However, the other study showed this association only among individuals with chronic kidney disease [61]. Thus, the mechanisms of the FGF23 role in CS-AMI is under debate. One of the possible pathophysiological reasons for the high levels of FGF23 in CS is the activation of RAAS and the sympathetic system, which may upregulate FGF-23 [59].

#### 3.8.5. Angiopoietin-2 

Angiopoietin-2 (Ang-2) is a growth factor belonging to one of the main pathways of angiogenesis; it is upregulated upon inflammatory stimuli and conditions such as hypoxia and cancer. An elevated concentration of Ang-2 is observed in coronary heart diseases, it is involved in cardiovascular remodeling, playing an important role in post-myocardial infarction recovery [62]. The substudy of the IABP-SHOCK II trial identified higher concentration of Ang-2 measured at the day of admission as an independent 30-day (HR 1.96; 95% CI 1.26–3.10; *p* = 0.002) and one-year (HR 2.21; 95% CI 1.49–3.27; *p* < 0.001) mortality prediction marker in CS-AMI patients [63]. In the IABP-SHOCK II Trial biomarker substudy, the Ang-2 tests carried out during the first 3 days have shown the increase of predictive value over time. The increase of Ang-2 was influenced by the baseline of Ang-2 along with AKI, bleeding events or transfusion, and impaired reperfusion. [63]. Moreover, the evaluation of critically ill patients with CS in the University Hospital of the Saarland, also showed Ang2 level above 2500 pg/mL as an independent predictor of 28-day and one-year mortality (HR 2.11; 95% CI 1.03–4.36; *p* = 0.042) [64]. 

Table 4 presents an overview of established and new biomarkers. 

## 4. Discussion

The studies of prognostic factors affecting the outcomes of CS are still continuing. In an attempt to predict outcomes and to identify high risk patients, risk scores including different clinical parameters were developed. However, the question “Are we at risk of having too many scores but too little information?”—posed by Dr. Teresa Lopez-Sobrino remains relevant [65]. Presented scoring systems have a number of limitations, some of the variables are to a certain extent subjective. A recently developed CLIP-scoring system predicting 30-day mortality in patients with CS-AMI is based on only four routinely available novel biomarkers and no manual scoring must be conducted [17]. This study has a number of limitations as well and a measurement bias cannot be excluded. However, this may be an example and inspiration for further studies based on the development of biomarker scoring systems that will be robust, easy to perform, and applicable in the heterogeneous population.

Knowing the prognostic role and mechanisms of the laboratory variables is important for the early detection of high-risk patients, both in the decision-making process in the management of cardiogenic shock, thus providing critically ill patients with better outcomes. 

## 5. Conclusions 

CS is the leading cause of death in patients with AMI. Despite the intensive development of varieties of new management approaches in the therapy of CS, the prognosis of patients with CS-AMI has remained for a long time without significant changes and improvement. Figuratively, every second patient with CS-AMI dies. The early identification of high-risk patients and well-timed indication for intensive care, including mechanical circulatory support, is essential. The use of laboratory parameters in risk-scoring systems seems to be a hopeful direction on this journey. An overview of knowledge about established and new biomarkers has the ambition to make an important contribution to existing knowledge in this field. Primary care clinicians may benefit from a summary of the pathogenic and prognostic role of the most relevant biomarkers in different stages of CS development after AMI, which may be fundamental in the risk stratification of this group of patients, Table 5. 

## Figures and Tables

**Table 1:** Risk stratification score systems of cardiogenic shock outcomes.

Study/Authors	Variables	Score Ranges and Predicted Mortality
IABP-SHOCK II trial [8]	Age > 73 years (1 point) Prior stroke (2 points) Glucose at admission > 10.6 mmol/L (1 point) Creatinine at admission > 132.6 μmol/L (1 point) Arterial blood lactate at admission > 5 mmol/L (2 points) TIMI flow grade < 3 after PCI (2 points)	30-day mortality risk Low (0–2)—28% Intermediate (3–4)—43% High (5–9)—77%
SHOCK trial [12]	Stage 1 (without invasive hemodynamics): Anoxic brain damage (30 points) Shock on admission (6 points) Noninferior myocardial infarction (3 points) Age (0–25 points) Clinical evidence of end-organ hypoperfusion (14 points) Prior CABG (7 points) Creatinine ≥ 1.9 mg/dL (5 points) Systolic blood pressure (6–12 points) Stage 2 (with invasive hemodynamics): Anoxic brain damage (25 points) Left ventricular ejection fraction < 28% (8 points) Age (0–20 points) End-organ hypoperfusion (14 points) Stroke Work g/m × (0–18 points)	In-hospital mortality risk at 30 days Without invasive hemodynamics: Range < 24 to ≥ 48 26–73% with early revascularization 23–91% with no/late revascularization With invasive hemodynamics: Range < 25 to ≥ 49 9–82% with early revascularization 19–85% with no/late revascularization
The GUSTO-I trial [14]	Without right heart catheterization: Age, height, baseline heart rate, blood pressure, time to thrombolytic treatment, prior infarction, prior angina, infarct location, Killip class, diabetes, smoking status, no extramyocardial factors, altered sensorium, cold clammy skin, oliguria, arrhythmia, ventricular septal defect, ventricular rupture. With right heart catheterization: Age, mean arterial pressure during shock, heart rate during shock, lowest cardiac output, highest pulmonary capillary wedge pressure,	30-day mortality risk (10–90%) Range 103–227 points without right heart catheterization Range 138–260 points with right heart catheterization
Cheng et al. [15]	Initial serum lactate (<1.7, 1.7–5.1, 5.1–8.5, >8.5) Age (<55, 55–65, 65–75, >75) Initial creatinine (above normal, >115 umol/L in men, >90 umol/L in women)	30-day mortality risk (8–89%) Two charts stratified by age, lactate (mmol/L), and serum creatinine (umol/L)
Garcia-Alvarez et al. [16]	Age > 75 years (1 point) Left main coronary occlusion (1 point) Left ventricular ejection fraction <25% (1 point) TIMI flow grade < 3 after PCI (1 point)	1-year survival risk Score 0–83% Score 1–19% Score > 2–6%
CLIP stratification score Ceglarek et al. [17]	Cystatin C Lactate Interleukin-6 NT pro brain natriuretic peptide	30-day mortality risk can be calculated from serum levels of these biomarkers, with the equation of the CLIP score

TIMI (thrombolysis in myocardial infarction), CABG (coronary artery bypass grafting), The GUSTO-I (Global Utilization of Streptokinase and Tissue Plasminogen Activator for Occluded Coronary Arteries) trial, Stroke Work = (MAP-PCWP) × 0.0136 × (CO/HR) × 1000 (where MAP = mean arterial pressure in mmHg, PCWP = pulmonary capillary wedge pressure in mmHg, CO = cardiac output, L/min, and HR = heart rate, bpm), CLIP stratification score (cystatin C, lactate, interleukin-6, and N-terminal pro-B-type natriuretic peptide).

**Table 2:** Mechanisms of effects of hyperglycemia on heart in CS-AMI.

Effects of Hyperglycemia	Mechanism
Metabolic derangement - higher levels of free fatty acids - insulin secretion - glycolysis - glucose oxidation Effects on myocardial ischemia and arrhythmias	Hyperglycemia in patients with STEMI is associated with higher concentrations of free fatty acid, myocardial glucose use impairment, and insulin resistance. These metabolic derangements are increasing consumption of oxygen leading to worsening of ischemia and resulting in acute heart failure development.
Promotion of inflammatory processes Prothrombotic effects	Increased release of inflammatory and vasoconstrictive factors leads to coronary endothelial dysfunction, contributing to oxidative stress and high platelet aggregation.

**Table 3:** Lactate blood concentration in prediction of developing cardiogenic shock in patients with myocardial infarction.

Predictive Value of Lactate in CS-AMI	No of Patients	Authors
Baseline lactate as well as change at 6, 12 and 24 h after admission is a powerful predictor of 30-day mortality	219	CardShock study [33]
Lactate above 6.5 mmol/L is an independent predictor of in-hospital mortality	45	Valente et al. [34]
Level of lactate along with creatinine and epinephrine dose is a significant univariate predictor of in-hospital mortality in patients with acute renal failure developed during the first 24 h of CS-AMI onset	118	Koreny et al. [35]
Admission lactate level above 3.8 mmol/L is a predictor of 30-day mortality	120	Lauten et al. [36]

**Table 4:** Laboratory predictors of cardiogenic shock outcomes.

**Established Biomarkers**			
**Laboratory Markers**	**Authors**	**No of Patients**	**Studied Prognostic Markers**
Serum creatinine level	Katz et al. [9]	396	Creatinine > 3.0 mg/dL (264 μmol/L)
	Klein et al. [10]	483	Creatinine > 2.0 mg/dL
	Bataille et al. [11]	2020	Creatine clearance < 60 mL/min
Glucose serum levels	Liu et al. [23]	7485	Hyperglycaemia
Serum lactate levels	Valente et al. [34]	45	Lactate > 6.5 mmol/L
	Koreny et al. [35]	118	Hyperlactatemie + acute renal failure
	Attana et al. [38]	51	Lactate clearance < 10%
	Lauten et al. [36]	120	Lactate level >3.8 mmol/L at admission
Hemoglobin concentration	Xu et al. [42]	211	Hemoglobin concentration < 112 g/L
Hypoalbuminemia	Jäntti et al. [43]	178	Hypoalbuminemia < 34 g/L
**Novel biomarkers**			
**Laboratory Markers**	**Authors**	**No of Patients**	**Studied Prognostic Markers**
Systemic inflammation markers: Interleukin-6,7,8,10 (IL-6,7,8,10) Interferon-gamma (INF-γ) Tumor necrosis factor alfa (TNF-α) Macrophage inflammatory protein-1β (MIP-1β) Granulocyte-colony stimulating factor (G-CSF) Monocyte chemoattractant protein-1β (MCP-1β)	Geppert et al. [50,51]	38	IL-6 > 200 pg/mL
	Prondzinsky et al. [52]	40	IL-8 (0.80 ± 0.08); IL-6 (0.79 ± 0.08); IL-10 (0.76 ± 0.08); IL-7 (0.69 ± 0.08)
	Prondzinsky et al. [53]	40	INF-γ, TNF-α, MIP-1β, G-CSF, and MCP-1β
	Fellner et al. [54]	58	Lower levels of activated protein C, inverse correlation with IL-6
Catalytic iron	Fuernau et al. [55]	600	High levels of catalytic iron
NT pro brain natriuretic peptide (NT-proBNP)	Radwan et al. [44]	560	High levels of NT-proBNP (>474 pg/mL)
	Jarai et al. [45]	58	Massive elevations of NT-proBNP (>12,782 pg/mL)
Osteoprotegerin (OPG) and Growth-differentiation factor 15 (GDF-15)	Fuernau et al. [57,58]	600	GDF-15 and OPG levels greater than the median
Fibroblast growth factor 23 (FGF-23)	Fuernau et al. [59]	600	FGF-23 levels above the median (395 RU/mL)
Angiopoietin-2 (Ang-2)	Pöss et al. [63]	600	High levels of Ang-2
	Link et al. [64]	1594	Ang-2 > 2500 pg/mL

**Table 5:** Proposes timing of biomarkers detection through the stages of cardiogenic shock.

Laboratory Biomarkers	Cardiogenic Shock Stage				
	A “At Risk“	B “Begining“	C “Classic“	D “Deteriorating“	E “Extremis“
Lactate	+	+	+	+	+
Creatinine clearence	+	+	+	+	+
Glucose serum level	+	+			
Hemoglobin	+	+	+		
Hypoalbuminemia	+				
NT-proBNP	+	+	+		
Systemic inflammation markers	+	+	+		
Catalytic iron	+				
OPG	+	+			
GDF-15	+	+			
FGF23	+	+			
Ang2	+	+			

NT-proBNP- NT pro brain natriuretic peptide, Systemic inflammation markers (interleukin-6,7,8,10, interferon-gamma, tumor necrosis factor alfa, macrophage inflammatory protein-1β, granulocyte-colony stimulating factor, monocyte chemoattractant protein-1β), OPG- osteoprotegerin, GDF-15-growth-differentiation factor 15, FGF23-fibroblast growth factor 23, Ang-2–angiopoietin-2.

## Data Availability

Not applicable.

## References

[1] Harjola V.-P., Lassus J., Sionis A., Køber L., Tarvasmäki T., Spinar J., Parissis J., Banaszewski M., Silva-Cardoso J., Carubelli V. (2015). Clinical picture and risk prediction of short-term mortality in cardiogenic shock: clinical picture and outcome of cardiogenic shock. Eur. J. Heart Fail..

[2] Hashmi K.A., Abbas K., Hashmi A.A., Irfan M., Edhi M.M., Ali N., Khan A. (2018). In-hospital mortality of patients with cardiogenic shock after acute myocardial infarction; impact of early revascularization. BMC Res. Notes.

[3] McDonagh T.A., Metra M., Adamo M., Gardner R.S., Baumbach A., Böhm M., Burri H., Butler J., Čelutkienė J., Chioncel O. (2021). 2021 ESC Guidelines for the diagnosis and treatment of acute and chronic heart failure: Developed by the Task Force for the diagnosis and treatment of acute and chronic heart failure of the European Society of Cardiology (ESC) with the special contribution of the Heart Failure Association (HFA) of the ESC. Eur. Heart J..

[4] Hochman J.S., Sleeper L.A., Webb J.G., Sanborn T.A., White H.D., Talley J.D., Christopher E.B., Jacobs A.K., Slater J.N., Col J. (1999). Early revascularization in acute myocardial infarction complicated by cardiogenic shock. SHOCK investigators. Should we emergently revascularize occluded coronaries for cardiogenic shock. N. Engl. J. Med..

[5] Thiele H., Zeymer U., Neumann F.-J., Ferenc M., Olbrich H.-G., Hausleiter J., Richardt G., Hennersdorf M., Empen K., Fuernau G. (2012). Intraaortic balloon support for myocardial infarction with cardiogenic shock. N. Engl. J. Med..

[6] Tehrani B.N., Truesdell A.G., Psotka M.A., Rosner C., Singh R., Sinha S.S., Damluji A.A., Batchelor W.B. (2020). A Standardized and Comprehensive Approach to the Management of Cardiogenic Shock. JACC. Heart Fail..

[7] Baran D.A., Grines C.L., Bailey S., Burkhoff D., Hall S.A., Henry T.D., Hollenberg S.M., Kapur N.K., O'Neill W., Ornato J.P. (2019). SCAI clinical expert consensus statement on the classification of cardiogenic shock. Catheter. Cardiovasc. Interv..

[8] Fuernau G., Poenisch C., Eitel I., Denks D., de Waha S., Pöss J., Heine G.H., Desch S., Schuler G., Adams V. (2015). Prognostic impact of established and novel renal function biomarkers in myocardial infarction with cardiogenic shock: A biomarker substudy of the IABP-SHOCK II-trial. Int. J. Cardiol..

[9] Katz J.N., Stebbins A.L., Alexander J.H., Reynolds H., Pieper K.S., Ruzyllo W., Werdan K., Geppert A., Dzavik V., Van de Werf F. (2009). TRIUMPH Investigators. Predictors of 30-day mortality in patients with refractory cardiogenic shock following acute myocardial infarction despite a patent infarct artery. Am. Heart J..

[10] Klein L.W., Shaw R.E., Krone R.J., Brindis R.G., Anderson H.V., Block P.C., McKay C.R., Hewitt K., Weintraub W.S. (2005). Mortality after emergent percutaneous coronary intervention in cardiogenic shock secondary to acute myocardial infarction and usefulness of a mortality prediction model. Am. J. Cardiol..

[11] Bataille Y., Déry J.-P., Larose É., Déry U., Costerousse O., Rodés-Cabau J., Gleeton O., Proulx G., Abdelaal E., Machaalany J. (2012). Deadly association of cardiogenic shock and chronic total occlusion in acute ST-elevation myocardial infarction. Am. Heart J..

[12] Sleeper L.A., Reynolds H., White H.D., Webb J.G., Džavík V., Hochman J. (2010). A severity scoring system for risk assessment of patients with cardiogenic shock: a report from the SHOCK Trial and Registry. Am. Heart J..

[13] Pöss J., Köster J., Fuernau G., Eitel I., de Waha S., Ouarrak T., Lassus J., Harjola V.P., Zeymer U., Thiele H. (2017). Risk stratification for patients in cardiogenic shock after acute myocardial infarction. J. Am. Coll Cardiol..

[14] Hasdai D., Holmes D.R., Califf R.M., Thompson T.D., Hochman J.S., Pfisterer M., Topol E. (1999). Cardiogenic shock complicating acute myocardial infarction: predictors of death. GUSTO Investigators. Global Utilization of Streptokinase and Tissue-Plasminogen Activator for Occluded Coronary Arteries. Am. Heart J..

[15] Cheng J.M., Helming A.M., Van Vark L.C., Kardys I., Uil C.A.D., Jewbali L.S.D., Van Geuns R.-J., Zijlstra F., Van Domburg R.T., Boersma E. (2016). A simple risk chart for initial risk assessment of 30-day mortality in patients with cardiogenic shock from ST-elevation myocardial infarction. Eur. Heart J. Acute Cardiovasc. Care.

[16] Garcia-Alvarez A., Arzamendi D., Loma-Osorio P., Kiamco R., Masotti M., Sionis A., Betriu A., Brugada J., Bosch X. (2009). Early risk stratification of patients with cardiogenic shock complicating acute myocardial infarction who undergo percutaneous coronary intervention. Am. J. Cardiol..

[17] Ceglarek U., Schellong P., Rosolowski M., Scholz M., Willenberg A., Kratzsch J., Zeymer U., Fuernau G., de Waha-Thiele S., Büttner P. (2021). The novel cystatin C, lactate, interleukin-6, and N-terminal pro-B-type natriuretic peptide (CLIP)-based mortality risk score in cardiogenic shock after acute myocardial infarction. Eur. Heart J..

[18] Ghionzoli N., Sciaccaluga C., Mandoli G., Vergaro G., Gentile F., D’Ascenzi F., Mondillo S., Emdin M., Valente S., Cameli M. (2020). Cardiogenic shock and acute kidney injury: The rule rather than the exception. Heart Fail. Rev..

[19] Tarvasmäki T., Haapio M., Mebazaa A., Sionis A., Silva-Cardoso J., Tolppanen H., Lindholm M.G., Pulkki K., Parissis J., Harjola V.-P. (2018). Acute kidney injury in cardiogenic shock: Definitions, incidence, haemodynamic alterations, and mortality. Eur. J. Heart Fail..

[20] Marenzi G., Assanelli E., Campodonico J., De Metrio M., Lauri G., Marana I., Moltrasio M., Rubino M., Veglia F., Montorsi P. (2010). Acute kidney injury in ST-segment elevation acute myocardial infarction complicated by cardiogenic shock at admission. Crit. Care Med..

[21] Singh S., Kanwar A., Sundaragiri P., Cheungpasitporn W., Truesdell A., Rab S., Singh M., Vallabhajosyula S. (2021). Acute Kidney Injury in Cardiogenic Shock: An Updated Narrative Review. J. Cardiovasc. Dev. Dis..

[22] Yap S.C., Lee H.T. (2012). Acute kidney injury and extrarenal organ dysfunction: new concepts and experimental evidence. Anesthesiology.

[23] Liu Y., Zhu J., Tan H.-Q., Liang Y., Liu L.-S., Li Y. (2010). Predictors of short term mortality in patients with acute ST-elevation myocardial infarction complicated by cardiogenic shock. Zhonghua Xin Xue Guan Bing Za Zhi.

[24] Pres D., Gasior M., Strojek K., Gierlotka M., Hawranek M., Lekston A., Wilczek K., Tajstra M., Gumprecht J., Poloński L. (2010). Blood glucose level on admission determines in-hospital and long-term mortality in patients with ST-segment elevation myocardial infarction complicated by cardiogenic shock treated with percutaneous coronary intervention. Kardiol. Pol..

[25] Vis M.M., Sjauw K.D., van der Schaaf R.J., Baan J., Koch K.T., DeVries J.H., Tijssen J.G., de Winter R.J., Piek J.J., Henriques J.P. (2007). In patients with ST-segment elevation myocardial infarction with cardiogenic shock treated with percutaneous coronary intervention, admission glucose level is a strong independent predictor for 1-year mortality in patients without a prior diagnosis of diabetes. Am. Heart J..

[26] Gokhroo R., Mittal S.R. (1989). Electrocardiographic correlates of hyperglycaemia in acute myocardial infarction. Int. J. Cardiol..

[27] Marfella R., De Angelis L., Siniscalchi M., Rossi F., Giugliano D., Nappo F. (2000). The effect of acute hyperglycaemia on QTc duration in healthy man. Diabetologia.

[28] Iwakura K., Ito H., Ikushima M., Kawano S., Okamura A., Asano K., Kuroda T., Tanaka K., Masuyama T., Hori M. (2003). Association between hyperglycaemia and the no-reflow phenomenon in patients with acute myocardial infarction. J. Am. Coll Cardiol..

[29] Malmberg K., Rydén L., Efendic S., Herlitz J., Nicol P., Waldenström A., Wedel H., Welin L. (1995). Randomized trial of insulin-glucose infusion followed by subcutaneous insulin treatment in diabetic patients with acute myocardial infarction (DIGAMI study): effects on mortality at 1 year. J. Am. Coll Cardiol..

[30] Malmberg K. (1997). Prospective randomised study of intensive insulin treatment on long term survival after acute myocardial infarction in patients with diabetes mellitus. DIGAMI (Diabetes Mellitus, Insulin Glucose Infusion in Acute Myocardial Infarction) Study Group. BMJ.

[31] Fuernau G., Desch S., de Waha-Thiele S., Eitel I., Neumann F.-J., Hennersdorf M., Felix S.B., Fach A., Böhm M., Pöss J. (2020). Arterial Lactate in Cardiogenic Shock: Prognostic Value of Clearance Versus Single Values. JACC Cardiovasc. Interv..

[32] Frydland M., Møller J.E., Wiberg S., Lindholm M.G., Hansen R., Henriques J.P., Møller-Helgestad O.K., Bang L.E., Frikke-Schmidt R., Goetze J.P. (2019). Lactate is a prognostic factor in patients admitted with suspected ST-elevation myocardial infarction. Shock.

[33] Lindholm M.G., Hongisto M., Lassus J., Spinar J., Parissis J., Banaszewski M., Silva-Cardoso J., Carubelli V., Salvatore D., Sionis A. (2020). Serum Lactate and a Relative Change in Lactate as Predictors of Mortality in Patients with Cardiogenic Shock-Results from the Cardshock Study. Shock.

[34] Valente S., Lazzeri C., Salvadori C., Chiostri M., Giglioli C., Poli S., Gensini G.F. (2007). Predictors of in-hospital mortality after percutaneous coronary intervention for cardiogenic shock. Int. J. Cardiol..

[35] Koreny M., Karth G.D., Geppert A., Neunteufl T., Priglinger U., Heinz G., Siostrzonek P. (2002). Prognosis of patients who develop acute renal failure during the first 24 hours of cardiogenic shock after myocardial infarction. Am. J. Med..

[36] Lauten A., Engström A.E., Jung C., Empen K., Erne P., Cook S., Windecker S., Bergmann M.W., Klingenberg R., Lüscher T.F. (2013). Percutaneous left-ventricular support with the Impella-2.5-assist device in acute cardiogenic shock: results of the Impella-EUROSHOCK-registry. Circ. Heart Fail..

[37] Englehart M.S., Schreiber M.A. (2006). Measurement of acid-base resuscitation endpoints: lactate, base deficit, bicarbonate or what?. Curr. Opin Crit. Care.

[38] Attaná P., Lazzeri C., Chiostri M., Picariello C., Gensini G.F., Valente S. (2012). Lactate clearance in cardiogenic shock following ST elevation myocardial infarction: a pilot study. Acute Card. Care.

[39] Marbach J.A., Stone S., Schwartz B., Pahuja M., Thayer K.L., Faugno A.J., Chweich H., Rabinowitz J.B., Kapur N.K. (2021). Lactate clearance is associated with improved survival in cardiogenic shock: a systematic review and meta-analysis of prognostic factor studies. J. Card Fail..

[40] Marbach J.A., Di Santo P., Kapur N.K., Thayer K.L., Simard T., Jung R.G., Parlow S., Abdel-Razek O., Fernando S.M., Labinaz M. (2022). Lactate Clearance as a Surrogate for Mortality in Cardiogenic Shock: Insights from the DOREMI Trial. J. Am. Heart Assos..

[41] Park I.H., Cho H.K., Oh J.H., Chun W.J., Park Y.H., Lee M., Kim M.S., Choi K.H., Kim J., Bin Song Y. (2021). Clinical Significance of Serum Lactate in Acute Myocardial Infarction: A Cardiac Magnetic Resonance Imaging Study. J. Clin. Med..

[42] Xu T., Liang D., Wu S., Zhou X., Shi R., Xiang W., Zhou J., Wang S., Shan P., Huang W. (2019). Association of hemoglobin with incidence of in-hospital cardiac arrest in patients with acute coronary syndrome complicated by cardiogenic shock. J. Int. Med. Res..

[43] Jäntti T., Tarvasmäki T., Harjola V.-P., Parissis J., Pulkki K., Javanainen T., Tolppanen H., Jurkko R., Hongisto M., Kataja A. (2019). Hypoalbuminemia is a frequent marker of increased mortality in cardiogenic shock. PLoS ONE.

[44] Radwan H., Selem A., Ghazal K. (2014). Value of N-terminal pro brain natriuretic peptide in predicting prognosis and severity of coronary artery disease in acute coronary syndrome. J. Saudi Heart Assoc..

[45] Jarai R., Fellner B., Haoula D., Jordanova N., Heinz G., Karth G.D., Huber K., Geppert A. (2009). Early assessment of outcome in cardiogenic shock: Relevance of plasma N-terminal pro-B-type natriuretic peptide and interleukin-6 levels. Crit. Care Med..

[46] Mega J.L., Morrow D.A., de Lemos J.A., Sabatine M.S., Murphy S.A., Rifai N., Gibson C., Antman E.M., Braunwald E. (2004). B-type natriuretic peptide at presentation and prognosis in patients with ST-segment elevation myocardial infarction: an ENTIRE–TIMI-23 substudy. J. Am. Coll Cardiol..

[47] Kohsaka S., Menon V., Lowe A.M., Lange M., Dzavik V., Sleeper L.A., Hochman J. (2005). Systemic inflammatory response syndrome after acute myocardial infarction complicated by cardiogenic shock. Arch. Intern. Med..

[48] Acharya D. (2018). Predictors of Outcomes in Myocardial Infarction and Cardiogenic Shock. Cardiol. Rev..

[49] Reynolds H.R., Hochman J.S. (2008). Cardiogenic shock: Current concepts and improving outcomes. Circulation.

[50] Geppert A., Steiner A., Zorn G., Delle-Karth G., Koreny M., Haumer M., Siostrzonek P., Huber K., Heinz G. (2002). Multiple organ failure in patients with cardiogenic shock is associated with high plasma levels of interleukin-6. Crit. Care Med..

[51] Geppert A., Dorninger A., Delle-Karth G., Zorn G., Heinz G., Huber K. (2006). Plasma concentrations of interleukin-6, organ failure, vasopressor support, and successful coronary revascularization in predicting 30-day mortality of patients with cardiogenic shock complicating acute myocardial infarction. Crit. Care Med..

[52] Prondzinsky R., Unverzagt S., Lemm H., Wegener N.-A., Schlitt A., Heinroth K.M., Dietz S., Buerke U., Kellner P., Loppnow H. (2012). Interleukin-6, -7, -8 and -10 predict outcome in acute myocardial infarction complicated by cardiogenic shock. Clin. Res. Cardiol..

[53] Prondzinsky R., Unverzagt S., Lemm H., Wegener N., Heinroth K., Buerke U., Fiedler G.M., Thiery J., Haerting J., Werdan K. (2012). Acute myocardial infarction and cardiogenic shock: prognostic impact of cytokines: INF-γ, TNF-α, MIP-1β, G-CSF, and MCP-1β. Med. Klin. Intensivmed. Notfmed..

[54] Fellner B., Rohla M., Jarai R., Smetana P., Freynhofer M.K., Egger F., Zorn G., Weiss T.W., Huber K., Geppert A. (2017). Activated protein C levels and outcome in patients with cardiogenic shock complicating acute myocardial infarction. Eur. Heart J. Acute Cardiovasc. Care.

[55] Fuernau G., Traeder F., Lele S.S., Rajapurkar M.M., Mukhopadhyay B., de Waha S., Desch S., Eitel I., Schuler G., Adams V. (2017). Catalytic iron in acute myocardial infarction complicated by cardiogenic shock—A biomarker substudy of the IABP-SHOCK II-trial. Int. J. Cardiol..

[56] Rochette L., Meloux A., Rigal E., Zeller M., Cottin Y., Vergely C. (2019). The Role of Osteoprotegerin and Its Ligands in Vascular Function. Int. J. Mol. Sci..

[57] Fuernau G., Poenisch C., Eitel I., de Waha S., Desch S., Schuler G., Adams V., Werdan K., Zeymer U., Thiele H. (2014). Growth-differentiation factor 15 and osteoprotegerin in acute myocardial infarction complicated by cardiogenic shock: a biomarker substudy of the IABP-SHOCK II-trial. Eur. J. Heart Fail..

[58] Fuernau G., Zaehringer S., Eitel I., de Waha S., Droppa M., Desch S., Schuler G., Adams V., Thiele H. (2013). Osteoprotegerin in ST-elevation myocardial infarction: prognostic impact and association with markers of myocardial damage by magnetic resonance imaging. Int. J. Cardiol..

[59] Fuernau G., Pöss J., Denks D., Desch S., Heine G.H., Eitel I., Seiler S., De Waha S., Ewen S., Link A. (2014). Fibroblast growth factor 23 in acute myocardial infarction complicated by cardiogenic shock: a biomarker substudy of the Intraaortic Balloon Pump in Cardiogenic Shock II (IABP-SHOCK II) trial. Crit. Care.

[60] Faul C., Amaral A.P., Oskouei B., Hu M.-C., Sloan A., Isakova T., Gutiérrez O.M., Aguillon-Prada R., Lincoln J., Hare J.M. (2011). FGF23 induces left ventricular hypertrophy. J. Clin. Invest..

[61] Agarwal I., Ide N., Ix J.H., Kestenbaum B., Lanske B., Schiller N.B., Whooley M.A., Mukamal K.J. (2014). Fibroblast growth factor-23 and cardiac structure and function. J. Am. Heart Assoc..

[62] Akwii R.G., Sajib M.S., Zahra F.T., Mikelis C.M. (2009). Role of Angiopoietin-2 in Vascular Physiology and Pathophysiology. Cells.

[63] Pöss J., Fuernau G., Denks D., Desch S., Eitel I., De Waha S., Link A., Schuler G., Adams V., Böhm M. (2015). Angiopoietin-2 in acute myocardial infarction complicated by cardiogenic shock-a biomarker substudy of the IABP-SHOCK II-Trial. Eur. J. Heart Fail..

[64] Link A., Pöss J., Rbah R., Barth C., Feth L., Selejan S., Böhm M. (2013). Circulating angiopoietins and cardiovascular mortality in cardiogenic shock. Eur. Heart J..

[65] Lopez-Sobrino T., Yusef H., Gershlick T. (2019). Predicting outcomes in cardiogenic shock: are we at risk of having too many scores but too little information?. Eur. Heart J..

